# Hippocampal function is not required for the precision of remote place memory

**DOI:** 10.1186/1756-6606-5-5

**Published:** 2012-02-02

**Authors:** Takashi Kitamura, Reiko Okubo-Suzuki, Noriko Takashima, Akiko Murayama, Toshiaki Hino, Hirofumi Nishizono, Satoshi Kida, Kaoru Inokuchi

**Affiliations:** 1 Department of Biochemistry, Faculty of Medicine, Graduate School of Medicine & Pharmaceutical Sciences, University of Toyama, Toyama 930-0194, Japan; 2 Japan Science and Technology Agency, CREST, Kawaguchi 332-0012, Japan; 3 Mitsubishi Kagaku Institute of Life Sciences, MITILS, Machida, Tokyo 194-8511, Japan; 4 Division of Animal Experimental Laboratory, Life Science Research Center, University of Toyama, Toyama 930-0194, Japan; 5 Department of Bioscience, Faculty of Applied Bioscience, Tokyo University of Agriculture, Tokyo 156-8502, Japan; 6 Present address: RIKEN-MIT Center for Neural Circuit Genetics and the Picower Institute for Learning and Memory, Massachusetts Institute of Technology, Cambridge, 02139, USA

## Abstract

**Background:**

During permanent memory formation, recall of acquired place memories initially depends on the hippocampus and eventually become hippocampus-independent with time. It has been suggested that the quality of original place memories also transforms from a precise form to a less precise form with similar time course. The question arises of whether the quality of original place memories is determined by brain regions on which the memory depends.

**Results:**

To directly test this idea, we introduced a new procedure: a non-associative place recognition memory test in mice. Combined with genetic and pharmacological approaches, our analyses revealed that place memory is precisely maintained for 28 days, although the recall of place memory shifts from hippocampus-dependent to hippocampus-independent with time. Moreover, the inactivation of the hippocampal function does not inhibit the precision of remote place memory.

**Conclusion:**

These results indicate that the quality of place memories is not determined by brain regions on which the memory depends.

## Introduction

The hippocampus is a key brain structure for learning and memory [1-3]. Recall of some associative and spatial memories initially depends on the hippocampus, but that hippocampal dependency progressively decays over time, a process that is associated with a gradual increase of neocortex-dependency [4-9]. It has been suggested that the quality of original memories also transforms from a precise (i.e., detailed) form to a less precise (i.e., more schematic or generic) form with similar time course [10-12]. The question arises of whether changes in the quality of the original memories depend on the shift in brain regions on which the recall of these memories relies, i.e., whether the hippocampus is always required for the precision of memories. This is an important question for understanding physiological significances of the hippocampal-cortical complementary memory systems.

There are several studies that address this issue in rodents. Using a contextual fear conditioning paradigm, which is an associative learning between a place and aversive experience, some studies demonstrated that the hippocampus is always necessary for the precision of place memories [13,14], supporting the memory transformation view [11] in which the quality of place memory correlates with the brain region on which that memory depends. By contrast, another study demonstrated that the hippocampus is not required for memory precision after the passage of time [15], supporting the memory reorganization view [9] in which the quality of place memory does not correlate with the brain region. Importantly, this discrepancy can be attributed to differences in experimental protocols used for association with fear [15]. Because association with fear modifies (i.e., strengthen or generalize) the precision [16,17], we cannot rule out the possibility that fear association may mask the actual precision of place memory. Moreover, contextual fear conditioning indirectly evaluates the place memory by measuring fear responses. Analyses based on association procedures may not be suitable for evaluating the precision. A direct way to address this issue is to measure the place memory *per se *without association protocols. In this study, we introduced a new procedure: a one-trial and non-associative place recognition test in mice. The clear advantage is the absence of any explicit reinforcement. In this procedure, we simply placed mice in a previously experienced vs. novel place. In the previously experienced place, mice exhibited habituation (reduced motility). In the novel context, mice did not show this reduction in motility. Thus, we evaluated the adaptation level as an index of the successful retrieval of place memory. Mice quickly acquired place memory during a free exploration and clearly discriminate experienced place from novel place. Especially mice maintained these memories for one month, which is different from the case in rats [18]. This procedure allows us to directly evaluate the precision of remote place memory. Using this procedure combined with genetic and pharmacological approaches, we examined the relationship between the precision of place memory and the brain region on which that memory depends.

## Materials & methods

### Animals

All animal procedures were conducted in compliance with the guidelines of the National Institutes of Health and were approved by the Animal Care and Use Committee of the University of Toyama and the Mitsubishi Kagaku Institute of Life Sciences. For WT experiments, male C57BL/6JSLC mice at 8 weeks of age were purchased from Sankyo Laboratory (Japan). For experiments using inducible cAMP response element binding protein (CREB) repressor transgenic mice [19,20] and α-Calcium-calmodulin kinase II heterozygous null mutant mice [21-23], the progeny for each line was produced using *in vitro *fertilization and embryo transfer techniques to produce a number of animals sufficient for behavioral testing. Age- and gender-matched littermates were used for the tests. All behavioral experiments were conducted and analyzed by scientists blind to the genotypes of the animals. Food and water were provided *ad libitum*.

### Cannulation and drug infusion

We used a surgical procedure described previously [24,25]. Briefly, mice were implanted bilaterally with stainless steel guide cannulae (Eicom) using the following stereotactic coordinates: AP = -2.0 mm, ML = ± 1.5 mm, V = -2.1 mm from the bregma. Mice were allowed to recover for at least 14 days in individual home cages. To transiently inactivate hippocampus, a fluorescently-labeled γ-aminobutyric acid subtype A receptor agonist (FCM, fluorophore-conjugated muscimol; Molecular Probe) was used. Mice were briefly anesthetized with isoflurane to facilitate the insertion of the injection cannula. FCM (0.8 mM, 0.5 ul) or PBS alone was infused into the dorsal hippocampus at a rate of 0.20 μl/min 60 min before the retrieval test. The fluorescent signals of FCM were distributed in the dorsal hippocampus bilaterally. To inhibit protein synthesis in the hippocampus, anisomycin (ANI, 100 μg/μl, 0.75 μl; Sigma) was infused into the dorsal hippocampus bilaterally at a rate of 0.25 μl/min 10 minutes after the learning. For the i.p. injection of ANI, ANI (150 mg/kg, i.p.) was dissolved in PBS (pH adjusted to 7.0-7.4) and administered to mice 10 minutes after the learning. At this dose, ANI inhibits 90% of protein synthesis in the brain during the first 2 hours [26]. For the i.p. injection of (R)-CPP (10 mg/kg, i.p.; Tocris), an antagonist of N-methyl-D-aspartate receptors, (R)-CPP was dissolved in PBS and administered to mice 4.5 hours before the learning. To examine the effects of disrupting CREB function on place memory formation, we used the transgenic mice that express an inducible CREB repressor in the forebrain, where a dominant-negative CREB protein is fused with the ligand binding domain of a mutant estrogen receptor [19]. Tamoxifen (4-hydroxytamoxifen; 16 mg/kg, i.p. TAM; Sigma), which was dissolved in 10 ml of peanut oil (Sigma), or peanut oil only (OIL) was administered to WT or transgenic mice 6 hours before the learning session.

### Place memory test

**Figure 1:**
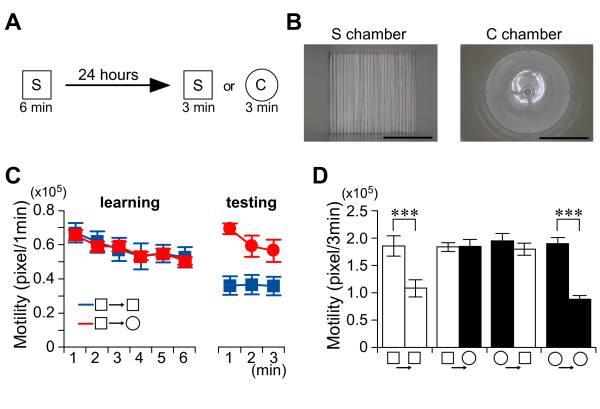
**Mice discriminate places in non-associative place recognition test**. *A*, Experimental design. *B*, Photographs of a square-type chamber and a circular-type chamber. Scale bars, 10 cm. *C*, Recent place memory test. Kinetics of motility in the learning and testing sessions. Blue line, S-S; red line, S-C; n = 6-7/group. *D*, Motility in the first 3 minutes during the learning or testing session in chamber S (white bars) or chamber C (black bars). n = 6-7/group. ***P < 0.001, **P < 0.01. Error bars indicate SEM.

Mice were housed individually in plastic cages with laboratory bedding at least 2 weeks before behavioral analyses and maintained on a 12:12 h light:dark cycle. Learning and testing sessions were conducted during the light cycle in a dedicated soundproof behavioral room (Room A: ASA4030, DR40, YAMAHA, JAPAN, (width × depth × height: 1640 × 1640 × 2160 mm, respectively) or other behavioral room (Room B: width × depth × height: 3200 × 4380 × 2240 mm, respectively). The square-type (S) chamber had a transparent acrylic-board front wall and gray side and back walls (width × depth × height: 175 × 165 × 300 mm, respectively), and the chamber floors consisted of 26 stainless steel rods with a diameter of 2 mm that were placed 5 mm apart. The circle-type (C) chamber is cylindrical (diameter × height: 170 × 270 mm, respectively) and has a white acrylic-board wall and floor (Figure 1B). During the learning session, mice were placed in the chamber (S or C chamber). After 6 minutes, mice were then returned to their home cages. During the testing session, mice were placed back into experienced chamber or the novel chamber for 3 minutes. At the end of each session, mice were returned to their home cages and the chambers were cleaned with water and 80% ethanol. All experiments were conducted using a video tracking system (Muromachi Kikai; Japan) to measure the motility of the animals. The motility was calculated as the cumulative area of movement (pixel size) per 0.1 sec in the learning and testing sessions.

### Contextual fear conditioning

Contextual fear conditioning was carried out as described previously [24,27,28]. Briefly, during the training session, mice were placed in the conditioning chamber (S) in Room A. After 3 minutes, animals were subjected to three unsignaled footshocks (2 sec duration, 0.5 mA, a min apart). After the last shock, mice remained in the chamber for 1 minute and were then returned to their home cages. During the testing session, mice were placed back into the conditioning chamber (S) or the novel chamber (C) for 3 minutes in Room A at 1 day or 28 days after training. At the end of each session, mice were returned to their home cages and the chambers were cleaned with water and 80% ethanol. All experiments were conducted using a video tracking system (Muromachi Kikai; Japan) to measure the freezing behavior of the animals. Freezing was defined as a complete absence of movement, except for respiration. Scoring of the duration of the freezing responses was started after 1 sec of sustained freezing behavior.

### Statistical analyses

All data are presented as mean ± SEM. The number of animals used is indicated by "n". Comparisons between two-group data were analyzed by unpaired Student's t-tests or paired t-tests. Multiple group comparisons were assessed using a one-way, two-way, or repeated measures analysis of variance (ANOVA), followed by the post-hoc Scheffe's test when significant main effects or interactions were detected. The null hypothesis was rejected at the *P *< 0.05 level.

## Results

Mice were exposed to a novel square-type (S) chamber to learn this place (Figure 1A, B). One day later, mice were again exposed to the same chamber or a novel circle-type (C) chamber to test memory retrieval (Figure 1A, C). We monitored the motility of mice in the chambers and evaluated the adaptation level for the experienced or novel chamber as an index of the successful retrieval of place memory (Figure 1C, D). Mice showed a reduction in their motility when exposed to the same S chamber (paired t-test; *t*_6 _= 9.96, *P *< 0.001) (Figure 1D). By contrast, a six-minute exposure to the S chamber did not induce adaptation in the novel C chamber (paired t-test; *t*_6 _= -0.07, *P *> 0.9) (Figure 1C, D). We obtained a similar result when we changed the sequence (C-C paired t-test: *t*_5 _= 8.39, *P *< 0.001; C-S paired t-test: *t*_6 _= 1.16, *P *> 0.2) (Figure 1D). These results indicate that mice quickly learn the place and completely discriminate the experienced place from the novel place one day after learning. Thus, the adaptation can be used to index place memory.

**Figure 2:**
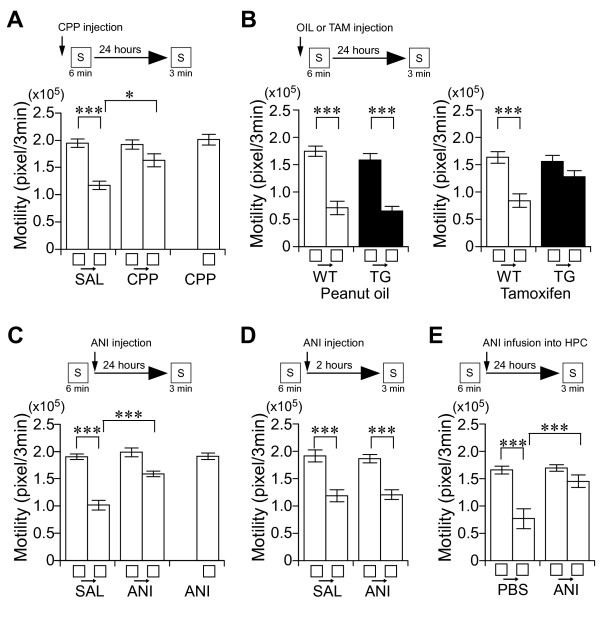
**Place memory formation requires NMDA receptor function, CREB-mediated transcription, and protein synthesis in the hippocampus**. *A*-*E*, Experimental design used with data presented below. *A*, Effect of an antagonist of the NMDA receptor on place memory formation. Motility in the first 3 minutes during the learning or testing session in chamber S. SAL (saline), CPP, or CPP treatment-only (homecage) groups. There was a significant interaction between adaptation and drug treatment (repeated ANOVA: F_(1, 47) _= 13.42, *P *< 0.01). n = 10-12/group. *B*, Effect of inducible repression of CREB function on place memory formation. Motility in the first 3 minutes during the learning or testing session in chamber S. There was a significant interaction between adaptation and genotype (repeated ANOVA: F_(1, 31) _= 7.32, *P *< 0.02). n = 6-9/group. *C*, Effect of i.p. injection of ANI on long-term place memory. Motility in the first 3 minutes during the learning or testing session in chamber S. SAL, ANI, or ANI treatment-only (homecage) groups. Injection of ANI disrupted long-term place memory. There was a significant interaction between adaptation and drug (repeated ANOVA: F_(1, 47) _= 15.89, *P *< 0.001). n = 10-12/group. *D*, Effect of i.p. injection of ANI on short-term place memory. Injection of ANI did not affect short-term place memory. n = 10/group. *E*, Effect of infusion of ANI into dorsal hippocampus on consolidation of place memory. There was a significant interaction between adaptation and drug (repeated ANOVA: F_(1, 21) _= 12.14, *P *< 0.001). n = 5-6/group. ***P < 0.001. **P < 0.01. *P < 0.05. Error bars indicate SEM.

To validate the potency of this procedure as place memory test, we have characterized the molecular mechanisms of place memory formation (Figure 2). We found that the injection of NMDA receptor antagonist disrupted the adaptation 1 day after learning (Figure 2A), and that the tamoxifen-injected CREB transgenic mice showed less adaptation for chamber S compared with the other groups (Figure 2B), and that an injection of anisomycin into the dorsal hippocampus disrupted the adaptation 1 day after learning (Figure 2E) but not short-term memory (Figure 2C, D). Thus, similar to other forms of hippocampus-dependent memory [3,29], the place memory formation requires the NMDA receptor function, the CREB-mediated transcription (Figure 2B), and the protein synthesis in the hippocampus.

**Figure 3:**
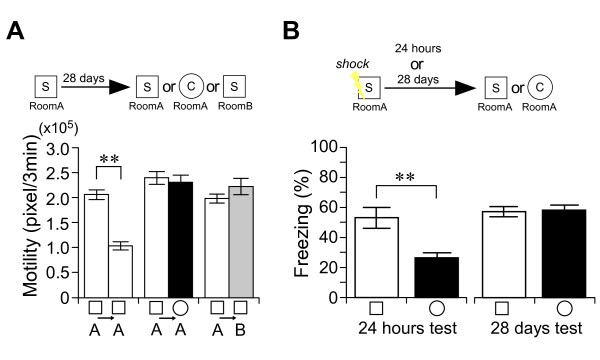
**The precision of non-associative place memory does not decline with time**. *A, B*, Experimental design used with data presented below. *A*, Remote place memory in non-associative place memory test. Motility in the first 3 minutes during the learning or testing session in chamber S/Room A (white bar), or chamber C/Room A (black bar), or chamber S/Room B (gray bar). There was a significant interaction between adaptation and chamber (chamber S/Room A vs chamber C/Room A; repeated ANOVA: F_(1, 23) _= 13.54, *P *< 0.01) (chamber S/Room A vs chamber S/Room B; repeated ANOVA: F_(1, 33) _= 31.86, *P *< 0.001). n = 6-11/group. *B*, Recent (1 day: n = 9-10/group) or remote (28 days: n = 19-20/group) contextual fear conditioning memory tests. Training was carried out in the S chamber. Freezing behavior averaged over the 3-minute test sessions in chamber S/Room A (conditioned, white bars) or chamber C/Room A (novel, black bars). There was a significant interaction between day and chamber (two-way ANOVA: F_(1, 58) _= 10.08, *P *< 0.01). ***P < 0.001, **P < 0.01. Error bars indicate SEM.

Next, we examined the maintenance of place memory 28 days after learning (Figure 3A). In the retrieval test, mice showed adaptation for the experienced S chamber (paired t-test: *t*_5 _= 6.48, *P *< 0.002), but not for the novel C chamber (paired t-test: *t*_5 _= 0.35, *P *> 0.7) (Figure 3A), indicating that mice can discriminate the experienced place from the novel place 28 days after learning. We further examined the precision of their remote place memory in a variable-rooms/constant-chamber condition, in which mice were placed for 6 minutes in the S chamber in room A and 28 days later these mice were placed in the same S chamber in a different room B (Figure 3A). In novel room B, mice did not show adaptation, even for the previously experienced S chamber (A-B, paired t-test: *t*_10 _= -1.69, *P *> 0.1) (Figure 3A), indicating that mice can detect changes outside of the chamber. This seems to be a similar feature of hippocampal representations, global remapping, in which both the firing location and firing rates of hippocampal pyramidal cells change in variable-room/constant-chamber conditions [30,31]. These results indicate that the precision of place memory does not decline with time in mice, even with one-trial learning. In marked contrast, mice seemed not to discriminate between the S chamber and the C chamber in a remote memory test for contextual fear conditioning (Figure 3B). Mice showed high freezing behavior in both chambers in the 28-day memory retrieval test (S vs C, *t*_37 _= -0.20, *P *> 0.8), although mice showed less freezing in the novel C chamber than in the conditioning S chamber in a one-day memory retrieval test (S vs C, *t*_17 _= 3.18, *P *< 0.01) (Figure 1E), as previously reported [13,15,24,32-34]. These results indicate that the fear association can mask the precision of place memory, whose mechanisms may be related to feed-forward inhibition growth in the hippocampus [34].

**Figure 4:**
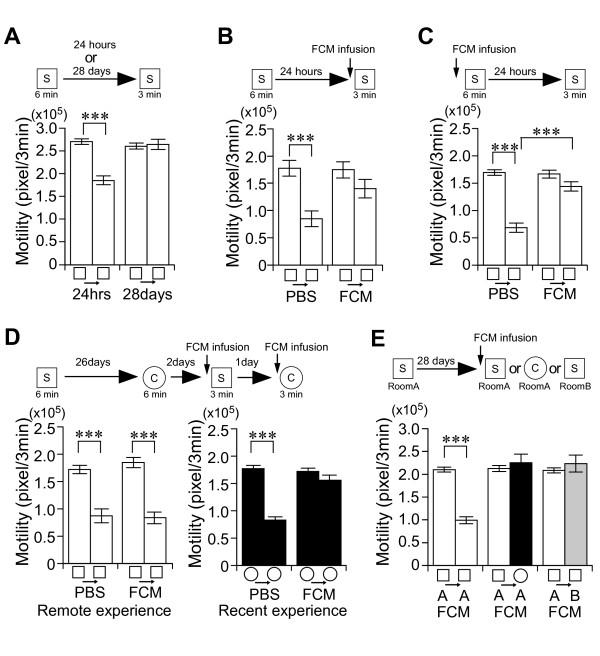
**The retrieval and precision of remote, but not recent, place memory does not require hippocampal function**. *A*-*E*, Experimental design used with data presented below. *A*, Recent and remote place memory in CaMK II heterozygous null mutant (HKO) mice. Motility in the first 3 minutes during the learning and testing session. There was a significant interaction between adaptation and day (repeated ANOVA: F_(1, 53) _= 36.55, *P *< 0.001). n = 13-14/group. *B, C*, Effect of the fluorescently-labeled muscimol (FCM) injection into the hippocampus on the formation (*C*) and the retrieval (*B*) of recent place memory. Motility in the first 3 minutes during the learning or testing session. There was a significant interaction between adaptation and drug (Figure 4B, repeated ANOVA: F_(1, 29) _= 23.61, *P *< 0.001) (Figure 4C, repeated ANOVA: F_(1, 33) _= 21.29, *P *< 0.001). *D*, Effect of FCM infusion into the hippocampus on the retrieval of remote and recent place memory. Chamber S was used for the remote memory retrieval test. Chamber C was used for the recent memory retrieval test. n = 10-11/group. E, Effect of the FCM infusion into the hippocampus on the retrieval of remote place memory in different chambers and rooms. Motility in the first 3 minutes during the learning or testing session. There was a significant interaction between adaptation and chamber (chamber S/Room A vs chamber C/Room A; repeated ANOVA: F_(1, 39) _= 35.71, *P *< 0.001) (chamber S/Room A vs chamber S/Room B; repeated ANOVA: F_(1, 43) _= 34.99, *P *< 0.001). n = 10-12/group. ***P < 0.001. Error bars indicate SEM.

Alpha-calcium-calmodulin kinase II (α-CaMKII) heterozygous null mutant (HKO) mice [21] have severe deficits in cortical long-term potentiation (LTP), whereas hippocampal CA1 LTP is normal [35]. The HKO mice showed high motility compared with age-matched litter mate wild-type mice (motility/3 min in Chamber S; WT (n = 10), 192876 **± **11743 pixel; HKO (n = 10), 274844 **± **15573 pixel; *t*_18 _= -4.2, *P *< 0.001), as previously reported [22]. Interestingly, HKO mice showed a complete deficit in place memory 28 days later (paired t-test: *t*_12 _= -0.36, *P *> 0.7), whereas one-day memory was normal (paired t-test; *t*_13 _= 9.09, *P *< 0.001) (Figure 4A). This suggests that cortical plasticity contributes to remote place memory formation. Here, we hypothesized that place memory temporally depends on the hippocampus. To test this, we assessed the contribution of the hippocampus to the retrieval of recent and remote place memories through the transient and pharmacological inactivation of the dorsal hippocampus (Figure 4B-D) [24]. The intrahippocampal infusion of fluorescently-labeled muscimol (FCM) [36] significantly inhibited the retrieval of 1-day memory (Figure 4B). The infusion of FCM also disrupted memory formation (Figure 4C). Treatment with FCM infusion alone did not affect natural motility (Figure 4B, C). Thus, hippocampal function is essential for the formation and retrieval of recent place memory. Next we examined the remote memory test. In the retrieval test for 28-day memory, both FCM- and phosphate buffered saline (PBS)-infused mice clearly showed adaptation to remotely experienced S chamber (PBS paired t-test: *t*_9 _= 6.30, *P *< 0.001; FCM paired t-test: *t*_10 _= 16.9, *P *< 0.001) (Figure 4D, *left panel*). There was no interaction between adaptation and drug treatment (repeated ANOVA: F_(1, 41) _= 0.35, *P *> 0.5). Subsequently, the same animals were subjected to recent memory retrieval test in which hippocampus was inactivated 60 min before the test by additional FCM infusion. The infusion of FCM significantly inhibited adaptation for the recently experienced C chamber (Figure 4D, *right panel*). There was a significant interaction between adaptation and drug treatment (repeated ANOVA: F_(1, 41) _= 27.1, *P *< 0.001), indicating that the FCM infusion effectively inactivated the hippocampal function in these animals. Therefore, the retrieval of remote place memory is completely hippocampal neuronal activity-independent.

Finally, we examined whether hippocampal function is required for the precision of remote place memory retrieval. In the retrieval test for 28-day memory, FCM-infused mice were placed in the experienced S chamber or novel C chamber (Figure 4E). FCM-infused mice showed adaptation for the experienced S chamber (paired t-test: *t*_9 _= 12.06, *P *< 0.001), whereas FCM-infused mice did not show adaptation for the novel C chamber (paired t-test: *t*_9 _= -0.68, *P *> 0.5) (Figure 4E). We further examined the variable-rooms/constant-chamber condition 28 days after learning (Figure 4E). Similar to naive mice (Figure 3A), FCM-infused mice did not show adaptation for the experienced S chamber in the novel room B (paired t-test: *t*_11 _= -0.84, *P *> 0.4) (Figure 4E). These results indicate that the pharmacological inactivation of hippocampal function does not inhibit the precision of remote place memory.

## Discussion

In this study, we carried out the contextual fear conditioning test and the non-associative place recognition test under the same experimental condition (same chambers and same exposure time to chamber) (Figure 3). These results clearly indicate that the association with fear masks the actual precision of place memory. Moreover, in contextual fear conditioning mice showed the freezing responses even for unconditioned place in recent memory test (Figure 3B) [13,15,24,32-34], whereas in our non-associative place recognition test mice did not show any adaptation behavior even for similar place in remote memory test (Figure 3A). Therefore, the conclusions of previous studies using contextual fear conditioning [13-15] need to be validated by non-associative protocol. Thus, we examined the contribution of hippocampal function on the precision of remote place memory by non-associative place recognition test.

Using this procedure, we found that the place memory is precisely maintained for 28 days, which may require α-CaMKII-dependent plasticity in the cortex, and that the retrieval of remote place memory (not recent memory) does not require hippocampal function. These results indicate that the quality of a place memory does not correlate with the brain region on which that memory depends. Moreover, we found that the inactivation of hippocampal function does not inhibit the precision of remote place memory. These results indicate that the hippocampal function is not required for the precision of remote place memory. This is consistent with a human case study in which a patient with bilateral extensive hippocampal damage showed intact memories for places learned long ago, but not intact recent place memory [37]. Eight patients with bilateral hippocampal damage were able to recall their remote autobiographical memories [38]. Thus, the quality of original place memories is not determined by brain regions on which the memory depends.

## Competing interests

The authors declare that they have no competing interests.

## Authors' contributions

T.K. and K.I. designed research; T.K., R.O.-S., N.T., A.M., T.H., H.N., S.K. performed research; T.K., R.O.S., and K.I. analyzed data; T.K., and K.I. wrote the paper; and K.I. supervised the entire project. All authors read and approved the final manuscript.

## References

[B1] Scoville WB Milner B Loss of recent memory after bilateral hippocampal lesions J Neurol Neurosurg Psychiatry 1957 20 11 21 10.1136/jnnp.20.1.11 PMC497229 13406589

[B2] Squire LR Stark CE Clark RE The medial temporal lobe Annu Rev Neurosci 2004 27 279 306 10.1146/annurev.neuro.27.070203.144130 15217334

[B3] Andersen P, Morris R, Amaral D, Bliss T, O'Keefe J The Hippocampus Book 2007 (New York: Oxford University Press)

[B4] Kim JJ Fanselow MS Modality-specific retrograde amnesia of fear Science 1992 256 675 677 10.1126/science.1585183 1585183

[B5] McClelland JL McNaughton BL O'Reilly RC Why there are complementary learning systems in the hippocampus and neocortex: insights from the successes and failures of connectionist models of learning and memory Psychol Rev 1995 102 419 457 10.1037/0033-295X.102.3.419 7624455

[B6] Dudai Y The neurobiology of consolidations, or, how stable is the engram? Annu Rev Psychol 2004 55 51 86 10.1146/annurev.psych.55.090902.142050 14744210

[B7] Wiltgen BJ Brown RA Talton LE Silva AJ New circuits for old memories: the role of the neocortex in consolidation Neuron 2004 44 101 108 10.1016/j.neuron.2004.09.015 15450163

[B8] Frankland PW Bontempi B The organization of recent and remote memories Nat Rev Neurosci 2005 6 119 130 10.1038/nrn1607 15685217

[B9] Squire LR Bayley PJ The neuroscience of remote memory Curr Opin Neurobiol 2007 17 185 196 10.1016/j.conb.2007.02.006 PMC2277361 17336513

[B10] Nadel L Moscovitch M Memory consolidation, retrograde amnesia and the hippocampal complex Curr Opin Neurobiol 1997 7 217 227 10.1016/s0959-4388(97)80010-4 9142752

[B11] Moscovitch M Nadel L Winocur G Gilboa A Rosenbaum RS The cognitive neuroscience of remote episodic, semantic and spatial memory Curr Opin Neurobiol 2006 16 179 190 10.1016/j.conb.2006.03.013 16564688

[B12] McKenzie S Eichenbaum H Consolidation and reconsolidation: two lives of memories? Neuron 2011 71 224 233 10.1016/j.neuron.2011.06.037 PMC3145971 21791282

[B13] Winocur G Moscovitch M Sekeres M Memory consolidation or transformation: context manipulation and hippocampal representations of memory Nat Neurosci 2007 10 555 557 10.1038/nn1880 17396121

[B14] Wiltgen BJ Zhou M Cai Y Balaji J Karlsson MG Parivash SN Li W Silva AJ The hippocampus plays a selective role in the retrieval of detailed contextual memories Curr Biol 2010 20 1336 1344 10.1016/j.cub.2010.06.068 PMC2928141 20637623

[B15] Wang SH Teixeira CM Wheeler AL Frankland PW The precision of remote context memories does not require the hippocampus Nat Neurosci 2009 12 253 255 10.1038/nn.2263 19182794

[B16] Houston FP Stevenson GD McNaughton BL Barnes CA Effects of age on the generalization and incubation of memory in the F344 rat Learn Mem 1999 6 111 119 PMC311284 10327236

[B17] Balogh SA Radcliffe RA Logue SF Wehner JM Contextual and cued fear conditioning in C57BL/6J and DBA/2J mice: context discrimination and the effects of retention interval Behav Neurosci 2002 116 947 957 10.1037//0735-7044.116.6.947 12492293

[B18] Izquierdo I da Cunha C Rosat R Jerusalinsky D Ferreira MB Medina JH Neurotransmitter receptors involved in post-training memory processing by the amygdala, medial septum, and hippocampus of the rat Behav Neural Biol 1992 58 16 26 10.1016/0163-1047(92)90847-w 1358054

[B19] Kida S Josselyn SA Pena de Ortiz S Kogan JH Chevere I Masushige S Silva AJ CREB required for the stability of new and reactivated fear memories Nat Neurosci 2002 5 348 355 10.1038/nn819 11889468

[B20] Kim R Moki R Kida S Molecular mechanisms for the destabilization and restabilization of reactivated spatial memory in the Morris water maze Mol Brain 2011 4 9 10.1186/1756-6606-4-9 PMC3045328 21314917

[B21] Silva AJ Wang Y Paylor R Wehner JM Stevens CF Tonegawa S Alpha calcium/calmodulin kinase II mutant mice: deficient long-term potentiation and impaired spatial learning Cold Spring Harb Symp Quant Biol 1992 57 527 539 10.1101/sqb.1992.057.01.058 1339689

[B22] Yamasaki N Maekawa M Kobayashi K Kajii Y Maeda J Soma M Takao K Tanda K Ohira K Toyama K Alpha-CaMKII deficiency causes immature dentate gyrus, a novel candidate endophenotype of psychiatric disorders Mol Brain 2008 1 6 10.1186/1756-6606-1-6 PMC2562999 18803808

[B23] Hasegawa S Furuichi T Yoshida T Endoh K Kato K Sado M Maeda R Kitamoto A Miyao T Suzuki R Transgenic up-regulation of alpha-CaMKII in forebrain leads to increased anxiety-like behaviors and aggression Mol Brain 2009 2 6 10.1186/1756-6606-2-6 PMC2660323 19257910

[B24] Kitamura T Saitoh Y Takashima N Murayama A Niibori Y Ageta H Sekiguchi M Sugiyama H Inokuchi K Adult neurogenesis modulates the hippocampus-dependent period of associative fear memory Cell 2009 139 814 827 10.1016/j.cell.2009.10.020 19914173

[B25] Kitamura T Saitoh Y Murayama A Sugiyama H Inokuchi K LTP induction within a narrow critical period of immature stages enhances the survival of newly generated neurons in the adult rat dentate gyrus Mol Brain 2010 3 13 10.1186/1756-6606-3-13 PMC2868842 20426820

[B26] Flood JF Rosenzweig MR Bennett EL Orme AE The influence of duration of protein synthesis inhibition on memory Physiol Behav 1973 10 555 562 10.1016/0031-9384(73)90221-7 4736141

[B27] Hayashi F Takashima N Murayama A Inokuchi K Decreased postnatal neurogenesis in the hippocampus combined with stress experience during adolescence is accompanied by an enhanced incidence of behavioral pathologies in adult mice Mol Brain 2008 1 22 10.1186/1756-6606-1-22 PMC2628657 19091092

[B28] Inoue N Nakao H Migishima R Hino T Matsui M Hayashi F Nakao K Manabe T Aiba A Inokuchi K Requirement of the immediate early gene vesl-1S/homer-1a for fear memory formation Mol Brain 2009 2 7 10.1186/1756-6606-2-7 PMC2663561 19265511

[B29] Lee YS Bailey CH Kandel ER Kaang BK Transcriptional regulation of long-term memory in the marine snail Aplysia Mol Brain 2008 1 3 10.1186/1756-6606-1-3 PMC2546398 18803855

[B30] Leutgeb S Leutgeb JK Barnes CA Moser EI McNaughton BL Moser MB Independent codes for spatial and episodic memory in hippocampal neuronal ensembles Science 2005 309 619 623 10.1126/science.1114037 16040709

[B31] Fyhn M Hafting T Treves A Moser MB Moser EI Hippocampal remapping and grid realignment in entorhinal cortex Nature 2007 446 190 194 10.1038/nature05601 17322902

[B32] Wiltgen BJ Silva AJ Memory for context becomes less specific with time Learn Mem 2007 14 313 317 10.1101/lm.430907 17522020

[B33] Biedenkapp JC Rudy JW Context preexposure prevents forgetting of a contextual fear memory: implication for regional changes in brain activation patterns associated with recent and remote memory tests Learn Mem 2007 14 200 203 10.1101/lm.499407 PMC2519802 17351145

[B34] Ruediger S Vittori C Bednarek E Genoud C Strata P Sacchetti B Caroni P Learning-related feedforward inhibitory connectivity growth required for memory precision Nature 2011 473 514 518 10.1038/nature09946 21532590

[B35] Frankland PW O'Brien C Ohno M Kirkwood A Silva AJ Alpha-CaMKII-dependent plasticity in the cortex is required for permanent memory Nature 2001 411 309 313 10.1038/35077089 11357133

[B36] Allen TA Narayanan NS Kholodar-Smith DB Zhao Y Laubach M Brown TH Imaging the spread of reversible brain inactivations using fluorescent muscimol J Neurosci Methods 2008 171 30 38 10.1016/j.jneumeth.2008.01.033 PMC2440580 18377997

[B37] Teng E Squire LR Memory for places learned long ago is intact after hippocampal damage Nature 1999 400 675 677 10.1038/23276 10458163

[B38] Bayley PJ Hopkins RO Squire LR Successful recollection of remote autobiographical memories by amnesic patients with medial temporal lobe lesions Neuron 2003 38 135 144 10.1016/s0896-6273(03)00156-9 12691671

